# Comparison of the Transcriptomes of Ginger (*Zingiber officinale* Rosc.) and Mango Ginger (*Curcuma amada* Roxb.) in Response to the Bacterial Wilt Infection

**DOI:** 10.1371/journal.pone.0099731

**Published:** 2014-06-18

**Authors:** Duraisamy Prasath, Raveendran Karthika, Naduva Thadath Habeeba, Erinjery Jose Suraby, Ottakandathil Babu Rosana, Avaroth Shaji, Santhosh Joseph Eapen, Uday Deshpande, Muthuswamy Anandaraj

**Affiliations:** 1 Indian Institute of Spices Research, Kozhikode (Calicut), Kerala, India; 2 Labindia-GPOD Research and Training Division, Thane, Maharashtra, India; University of North Carolina at Charlotte, United States of America

## Abstract

Bacterial wilt in ginger (*Zingiber officinale* Rosc.) caused by *Ralstonia solanacearum* is one of the most important production constraints in tropical, sub-tropical and warm temperature regions of the world. Lack of resistant genotype adds constraints to the crop management. However, mango ginger (*Curcuma amada* Roxb.), which is resistant to *R. solanacearum*, is a potential donor, if the exact mechanism of resistance is understood. To identify genes involved in resistance to *R. solanacearum*, we have sequenced the transcriptome from wilt-sensitive ginger and wilt-resistant mango ginger using Illumina sequencing technology. A total of 26387032 and 22268804 paired-end reads were obtained after quality filtering for *C. amada* and *Z. officinale*, respectively. A total of 36359 and 32312 assembled transcript sequences were obtained from both the species. The functions of the unigenes cover a diverse set of molecular functions and biological processes, among which we identified a large number of genes associated with resistance to stresses and response to biotic stimuli. Large scale expression profiling showed that many of the disease resistance related genes were expressed more in *C. amada*. Comparative analysis also identified genes belonging to different pathways of plant defense against biotic stresses that are differentially expressed in either ginger or mango ginger. The identification of many defense related genes differentially expressed provides many insights to the resistance mechanism to *R. solanacearum* and for studying potential pathways involved in responses to pathogen. Also, several candidate genes that may underline the difference in resistance to *R. solanacearum* between ginger and mango ginger were identified. Finally, we have developed a web resource, ginger transcriptome database, which provides public access to the data. Our study is among the first to demonstrate the use of Illumina short read sequencing for *de novo* transcriptome assembly and comparison in non-model species of Zingiberaceae.

## Introduction

Ginger (*Zingiber officinale* Rosc.) is a widely used spice, flavoring agent, herbal medicine and is also employed in the perfume industry. Cultivated ginger is a sterile crop, originated in India or Southeast Asia [1]. Today ginger is cultivated in many tropical and subtropical areas. The main producers are India, China, Indonesia, and Nigeria [2]. Ginger is susceptible to many diseases. Among those, bacterial wilt (*Ralstonia solanacearum*) is one of the most important production constraints in tropical, sub tropical and warm temperature regions of the world [3]. Geographical distribution of the pathogen is expanding in recent years due to unintentional transmission of the bacterium through infected rhizomes of ginger, which are the primary propagules [4]. In spite of extensive search, no resistance source to *Pythium* soft rot, *Fusarium* yellows and *Ralstonia* induced bacterial wilt could be located in *Zingiber* genus. This is due to lack of genetic variability among the accessions for disease resistance, which is one of the bottlenecks in ginger genetic improvement. Resistance breeding in ginger is restricted to germplasm screening as it is an obligatory asexual crop [5]. The search for resistance has been extended to other closely related genera in the family, Zingiberaceae such as *Curcuma amada, C. longa*, *C. zedoaria, C. aromatica, Kaempferia galanga, Elettaria cardamomum, Zingiber zerumbet* and *Z. officinale* for their reaction to *R. solanacearum* biovar 3 (ginger specific strain) and *Pythium* species. The Indian mango ginger (*Curcuma amada* Roxb.) exhibited significant resistance to both the pathogens [6], [7], while *Z. zerumbet* is resistant to *P. aphanidermatum* [8]. The high level of resistance recorded in *C. amada* to *Ralstonia* wilt is providing an opportunity for developing bacterial wilt resistance in ginger. However, this can be done only if the mechanism of resistance in *C. amada* is known. A thorough genetic analysis would unravel the factors (genes) governing the resistance in *C. amada*–*R. solanacearum* pathosystem [6]. Lack of genetic variability in ginger coupled with available resistance in a closely related genus makes the use of functional genomics an ideal choice to impart *R. solanacearum* resistance in ginger.

Development of genomic tools will certainly facilitate the isolation of resistance genes and provide genetic reagents for developing resistant varieties by genetic engineering. The absence of seed set in ginger makes conventional breeding methods inapplicable warranting genetic modification through biotechnological means. Ginger is transformable using *Agrobacterium tumefaciens* [9] and methods for plant regeneration from somatic embryos have been developed [10]–[12], permitting the production of many individuals from single transformation events.

Expressed sequence tag (EST) sequencing has traditionally been the core technology used for the discovery of reference transcripts. However, it has some inherent limitations, such as low throughput, high cost and a long experimental cycle. Recently, researchers have developed a high-throughput sequencing technology called Next generation sequencing (NGS) [13]. Various platforms utilize NGS, such as the Illumina Genome Analyzer, the Roche/454 Genome Sequencer FLX Instrument, and the ABI SOLiD System; these have proven to be powerful and cost-effective tools for advanced research in many areas, including genome sequencing, genome resequencing, miRNA expression profiling, DNA methylation analysis, and especially the *de novo* transcriptome sequencing of non-model organisms [14], [15]. This method of transcriptome analysis is fast and simple because it does not require bacterial cloning of the cDNAs. Instead, direct cDNA sequencing generates an extraordinary depth of short reads. It is a more comprehensive and efficient way to measure transcriptome composition, obtain RNA expression patterns, and discover new genes. In addition, this approach is very sensitive, and thus allows the detection of low-abundance transcripts. Illumina genome analyzer based sequencing technology (Illumina, San Diego, CA, USA) yields huge amount of short reads with high coverage. Assembling such short reads is a challenging task, more so in the absence of reference sequences. A few bioinformatics tools have been developed for *de novo* assembly using short-read sequence data [16], which vary in their success and application, and depends upon data specific strategies.

The work reports a strategy for *de novo* assembly of transcriptome using short-read sequence data generated by Illumina RNA-Seq method. A comparison of capillary sequencing and next generation sequencing methods showed that short-read sequencing is well adapted for analyzing the transcriptome of both model and non-model species, with lower cost than conventional methods such as microarrays, Serial analysis of gene expression (SAGE) or EST analysis generated using capillary sequencing. Recent transcriptomic studies on *Arabidopsis thaliana* [17-], and many non-model plants (*Cicer arietinum* L. [18], *Daucus carota* var. *sativus* L. [19], *Hevea brasiliensis* [20], *Picrorhiza kurrooa* Royle ex Benth [21], *Carthamus tinctorius* L [22], *Costus pictus* D. Don [23], *Allium sativum* [24], *Raphanus sativus* L. [25], *Piper nigrum* L. [26], [27], *Curcuma longa* L. [28], *Saccharum officinarum L.* [29]) have demonstrated that this approach is well-suited for surveying the complexity of eukaryotic transcriptomes by *de novo* assembly.

ESTs are considered to represent a reliable source of data for predicting microRNAs (miRNAs) and their targets, especially in species without complete genome information [30]. miRNAs are important regulators in a wide range of developmental processes in plants, including cell proliferation, the stress response, metabolism, inflammation and signal transduction [31]. Evolutionarily conserved targets have been shown to be useful in testing the effectiveness of miRNA target detection. A perfect, or near perfect, complementarity between an miRNA and its target mRNA, which is a peculiar feature of plant miRNAs, provides a powerful tool for the identification of target genes through BLAST analysis of mature miRNA sequences against EST sequences. However, either miRNAs or their target genes have not yet been identified from *C. amada* and *Z. officinale*.

Genomic tools are now being developed to accelerate the identification of resistance genes and the development of bacterial wilt resistant ginger. In this context, a central objective is the sequencing of the transcriptomes of ginger and mango ginger species with a long-term goal of isolating genes underlying resistance to the bacterial wilt. The present study describes the first global analysis of ginger and mango ginger - *R. solanacearum* challenge inoculated regimes, which would serve as a blueprint of gene expression profile. Comparative transcriptomics approach was adopted to systematically characterize the mRNAs to identify the differentially regulated genes, including those involved in disease resistance. The comparison between the ginger and mango ginger transcriptomes enabled us to identify a large number of candidate pathogen response genes for use in studying pathways involved in resistance to the bacterial wilt.

## Materials and Methods

### Ginger and Mango Ginger Materials

Healthy rhizomes of ginger and mango ginger were collected from National Active Germplasm Site (NAGS) at the Indian Institute of Spices Research (IISR), Experimental Farm, Peruvannamuzhi, Kerala, India. Forty five to 60 days old *C. amada* and *Z. officinale* plants grown in the greenhouse of the IISR (Kozhikode, Kerala, India) was used for the experiment. The virulent colonies of *R. solanacearum*, as identified on Casamino acid-Peptone-Glucose (CPG) medium [32], were multiplied in sucrose peptone broth (g l^−1^ sucrose, 20; peptone, 10; K_2_HPO_4_, 0.5; MgSO_4_, 0.25; pH 7.2) for two days. The bacteria was pelleted at 10000 g for 20 min at 4°C, resuspended in water and poured around the base of the 45-day-old plants, as water suspension at a concentration of 10^9^ cells ml^−1^ of water. The inoculated plants were grown in greenhouse (28±2°C, 12 h light, 65% RH) and were monitored for wilt disease. Leaf tissues were sampled over an 72 h period post-inoculation and pooled before RNA preparation. All samples were collected, frozen in liquid nitrogen and stored at −80°C until use.

### RNA Isolation

Total RNA Was Isolated Using the Trizol Reagent (Invitrogen, Carlsbad, CA, USA) According to the Manufacturer’s Instructions. the RNA Samples Were Treated with 10 Units of Dnasei (Takara, Dalian, China) for 30 Min at 37°C to Remove the Genomic DNA. the Total RNA Quality Was Verified Using RNA 6000 Nano Kit (Agilent Technologies, Santa Clara, CA, USA) on 2100 Bioanalyzer (Agilent Technologies, Santa Clara, CA, USA), with a Minimum RNA Integrity Number (RIN) Value of 8.

### Preparation of Library and Sequencing

Two paired-end RNA-Seq libraries were generated, one each from total RNA extracted from ginger and mango ginger tissues. The RNA-Seq library construction and sequencing was performed by commercial service provider (NxGenBio Life Sciences, New Delhi, India). Total RNA was used to enrich mRNA using Oligotex mRNA midi prep kit (QIAGEN, Hilden, Germany) from two µg of total RNA using oligo (dT) magnetic beads and fragmented into 200–500 bp pieces using divalent cations at 94°C for 5 min. The cleaved RNA fragments were copied into first strand cDNA using SuperScript II reverse transcriptase (Life Technologies, Carlsbad, CA, USA) and random primers. After second strand cDNA synthesis, fragments were end repaired and A-tailed. The cDNA libraries were constructed for the ginger and mango ginger using the TruSeq RNA sample preparation kit (Illumina) with alternate fragmentation method for generating 200–500 bp fragments, according to manufacturers’ instructions. The tagged Paired-End RNA-Seq libraries were diluted and pooled in equimolar concentrations and sequenced using TruSeq SBS kit V3 on HiSeq2000 (Illumina) for generating 2×100 bp sequencing reads. The raw reads generated for mango ginger and ginger were 64.33 and 48.70 millions respectively, with more than 90% of the bases having a phred score of Q20.

### Transcriptome Assembly

The raw reads generated for mango ginger and ginger were subjected to *de novo* assembly using the CLC Genomics Workbench ver. 6.0 (CLC Bio, Swansea, UK). Reads were also subjected to quality check using the parameters like per-sequence analysis, per-base analysis and over representation analysis. Further reads were filtered for ambiguity, low quality and PCR duplicates. Total clean reads obtained for mango ginger and ginger were 26387032 and 22268804, respectively and subjected to further downstream analysis.

*De novo* transcriptome assembly was performed using CLC bio Genomic Workbench ver. 6.0, with the default settings *k*-mer size of 25, minimum contig length of 300, paired fragment length of 500 with parameters (insertion/deletion cost = 3, mismatch cost = 2, 80% of read length with similarity of 90%) for both the assemblies using the clean reads.

### Functional Annotation

Functional annotation of the generated contigs was performed using Blast2GO software [33]. Using default settings of Blast2GO, contig sequences were searched against the NCBI non-redundant protein database (nr) with BLAST expectation value of 1.0e-3 and maximum 20 hits, HSP length cutoff (default = 33) with low complexity filter on was used. Mapping step involved retrieval of Gene Ontology (GO) terms associated with each BLAST hit. GO annotation step assigns GO annotation to the query sequence with annotation score parameters; e-value hit filter (default = 1.0E-6), annotation cut-off (default = 55), GO-weight (default = 5), hsp-hit coverage cut off (default = 0). Additionally, conserved domains/motifs using InterProScan were extracted using a plug-in within the Blast2GO. Blast2Go also retrieved Kyoto Encyclopedia of Genes and Genomes (KEGG) maps for the query sequence. We have also used FastAnnotator online tool (http://fastannotator.cgu.edu.tw), which performs annotations utilizing Blast2GO and PRIAM to identify GO terms and EC numbers for transcript sequences [34].

### Measurement of Gene Expression

Gene expression analysis was performed by mapping the raw reads back to the *de novo* transcriptome assemblies of ginger and mango ginger, using RNA-Seq mapping tools of CLC Genomic Workbench ver 6.0. We also mapped clean reads to *de novo* transcriptome assembly of mango ginger. RNA-Seq read mapping parameters were insertion cost 2, deletion cost 2, mismatch cost 3, similarity 90%, with unique mapping of reads to a single location. List of differentially expressed gene between ginger and mango ginger was obtained using analysis tools in CLC bio Genomic Workbench ver 6.0. The fold change in expression was calculated based on number of reads mapped and RPKM. Kal’s Z test statistics was applied to test significance of fold change at P<0.005 and FDR at 0.05%.

### Target Prediction for the *C. amada* miRNAs

Growing evidences have shown that most plant miRNAs function by either perfectly or near-perfectly binding to complementary sites on their target mRNA sequences [35]. This provides a powerful way to identify potential miRNA targets by aligning and comparing miRNAs with potential target sequences. The *C. amada* and *Z. officinale* assembled contigs were submitted to psRNATarget webserver [36] for predicting miRNA targets. In this research, the following default parameters were used for identifying potential miRNA targets: (1) maximum expectation 3; (2) length for complementarity scoring (hspsize) 20; (3) target accessibility - allowed maximum energy to unpair the target site (UPE) 25; (4) flanking length around target site for target accessibility analysis 17 bp upstream and 13 bp downstream and (5) range of central mismatch leading to translational inhibition 9–11 nucleotides.

### Construction of Database

gTDB is a public resource for ginger and mango ginger transcriptome data. Web pages have been prepared using HTML, PHP as frontend and MySQL as backend with WAMP server application. The data regarding expression and annotation for each transcript are stored in the MySql server. The database is currently hosted on http://220.227.138.212/GTDB/. The sequence data are stored in flat files.

## Results

### Illumina Paired-end Sequencing and Sequence Quality Control

In order to achieve a broad survey of genes associated with bacterial wilt resistance, we performed mRNA-Seq profiling of *C. amada* and *Z. officinale* leaves, following infection with *R. solanacearum* and the resulting sequencing data were subjected to bioinformatic analysis. A total of 31845321×2 (101 base) and 24107482×2 (101 base) raw reads, accounting for approximately 6.43 Gb and 4.87 Gb of sequence data, for *C. amada* and *Z. officinale*, respectively were generated. The difference in the number of reads generated for the *C. amada* and the *Z. officinale* reflects the lower quality of the *Z. officinale* library. After cleaning and quality checks, a total of 56.09 million and 42.39 million clean reads with 88.07 and 87.91% Q20 bases (base quality more than 20) were generated from the *C. amada* and *Z. officinale* cDNA libraries, respectively (Table 1). After removing the adapter, low quality sequences and PCR duplicates from the raw data, 26.38 million and 22.26 million high quality reads were retained for *C. amada* and *Z. officinale,* respectively. These high quality, processed paired-end reads were used to assemble into contigs and further into transcripts.

**Table 1:** Assembly summary of *C. amada* and *Z. officinale.*

	*C. amada*	*Z. officinale*
Total nucleotides (Nt)	6432754842	4869711364
Total number of clean reads	56090429	42386900
Q20 percentage	88.07	87.91
GC precentage	47	47
Total number of reads after PCR duplicates	26387032	22268804
Number of contigs	36359	32312
Maximum contig length	13664	12817
Minimum contig length	300	300
Total contig length (bases)	27331488	25350555
Mean contig length	752	785
N75	536	570
N50	872	943
N25	1453	1481

### Transcriptome Assembly

**Figure 1:**
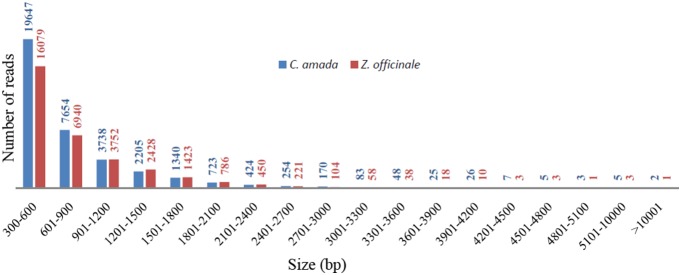
Sequence length distribution of assembled contigs in the transcriptomes of C. amada and Z. officinale. Histogram presentation of sequence-length distribution for significant matches that was found. The x-axis indicates sequence sizes from 300 nt to >10001 nt. The y-axis indicates the number of contigs for every given size range.

The results of *de novo* transcriptome assembly are summarized in Table 1. The *C. amada* assembly was represented by 36359 contigs and its length ranged from 300 to 13664 bps with average of 752 bps, while *Z. officinale* assembly was represented by 32312 contigs with maximum contig size of 12817 bps and minimum contig size of 300 bps (Figure 1). The N50 for *C. amada* and *Z. officinale de novo* assemblies were 872 and 943, respectively. Both transcriptome assemblies found to be comparable based on the assembly statistics. These assemblies were further annotated with Blast2GO tool and used for the RNA-Seq mapping for differential expression analysis.

### Functional Annotation and Classification

**Figure 2:**
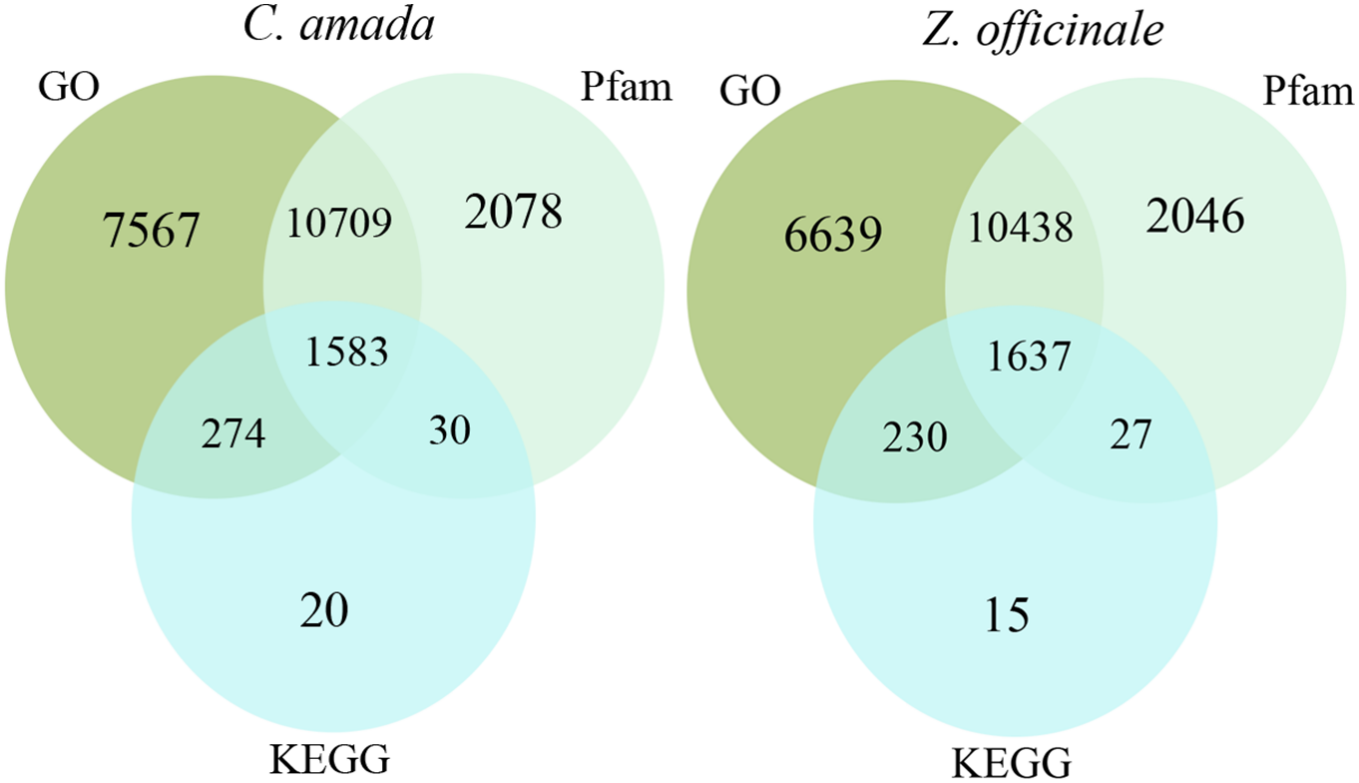
Distribution of similarity search results showed by Venn diagrams. The number of unique sequence-based annotations is the sum of unique best BLASTX hits from the GO term, Pfam domains and KEGG databases, respectively. The overlap regions among the three circles contain the number of unigenes that share BLASTX similarity with respective databases.

Functional annotation of novel plant transcriptomes is a difficult task due to the limited availability of reference genome/gene sequences in public databases. Being a non-model plant and without much availability of reference sequences in the databases, it is challenging to predict accurate annotations for the transcripts. Blast2GO annotated 73.17% of *C. amada* contigs and 76.55% of *Z. officinale* contigs. In case of *C. amada,* 7569 contigs were annotated with unique GO terms, 2078 with unique Pfam domains, 20 with unique KEGG enzymes. Whereas, 10709 contigs were annotated with GO terms and Pfam domain jointly, 1583 contigs were annotated with GO term, Pfam domain and KEGG enzyme overlapping annotations (Figure 2). Additionally, 4343 contigs were annotated with gi accession number form the NCBI GenBank. Thus out of 36359 total contigs in *C. amada,* 26606 contigs were annotated with at least one type of annotation. Similarly in *Z. officinale*, 6639 contigs were annotated with unique GO terms, 2046 contigs with unique Pfam domains, 15 contigs with unique KEGG enzymes. Additionally, 230 contigs with GO term and KEGG enzyme overlapping, 10438 with GO term and Pfam domain over lapping terms and 1637 with three overlapping terms were annotated (Figure 2). Additionally 3706 contigs were annotated with gi accession number from the NCBI GenBank. Thus out of 32312 total contigs, 24738 contigs were annotated in *Z. officinale.*

Gene Ontology (GO) analysis [37] showed that the distributions of gene functions for cDNA sequences from *C. amada* and *Z. officinale* were almost similar. This expected result indicates that there is no bias in the construction of the libraries from both species. Based on different kinds of functional categories, the biological process made up majority followed by cellular component and molecular function. In *C. amada,* 15092 biological processes, 15454 cellular components, 16961 molecular functions, 14210 Pfam domains, and 1907 KEGG enzymes annotations were associated with annotated unigenes. In case of *Z. officinale,* 11443 biological processes, 11784 cellular components, 12911 molecular functions, 11498 Pfam domains and 1620 enzymes were associated with annotated unigenes. After annotation clustering was performed to identify the contigs being assigned similar annotation.

**Figure 3:**
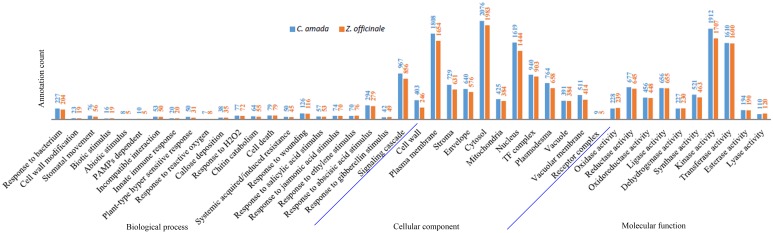
Histogram presentation of Gene Ontology classification of assembled contigs. The results are summarized in three main categories: biological process, cellular component and molecular function. The y-axis indicates the number of genes in a category.

Classified GOs were further grouped using terms associated with the defense transcriptome of the two species. It is noteworthy that a larger number of genes involved in response to biotic and abiotic stimuli and stresses were identified in *C. amada* compared with *Z. officinale*. The highest numbers of GO terms were assigned to the term response to bacterium followed by response to wounding and cell death (Figure 3). The Salicylic acid, jasmonic acid, ethylene, gibberellic acid and abscisic acid mediated signaling were also represented in both the transcriptomes. The overall distribution of GO biological process exhibited more annotations of *C. amada* than *Z. officinale*. This difference may be associated with bacterial wilt resistance in *C. amada*. The GO terms related to molecular function exhibited highest annotations for kinase activity followed by transferase activity, reductase, transporter and hydrolase activity (Figure 3). The cytosol, plasma membrane, and nucleus received the highest number of GO cellular component annotations followed by TF complex, plasmodesma and, vacuole (Figure 3). *C. amada* registered marginally higher assignments compared to *Z. officinale*.

### Analysis of the *C. amada* and *Z. officinale* Transcriptomes

BLASTX searches were then conducted against the proteomes of all of the plant species for which the whole genome sequence were available at the time of this study, including *Arabidopsis thaliana*, *Vitis vinifera, Carica papaya, Oryza sativa, Populus trichocarpa* and *Selaginella moellendorffii.* About 68.0% and 69.4% of predicted proteins of *C. amada* and *Z. officinale* had a significant homology (limit of 1e^–05^) with *A. thaliana* proteins sequences in the NCBI database. The species with the highest hit rates were *V. vinifera* with 71.3% and 72.6%, *C. papaya* with 70.0% and 71.7% and *P. trichocarpa* with 69.2% and 70.5% of hits for *C. amada* and *Z. officinale*, respectively (Figure S1). The BLASTX species hit statistics revealed that more than 30 plant species served as a source for the annotations.

### Transcriptome Comparison between *C. amada* and *Z. officinale*

**Figure 4:**
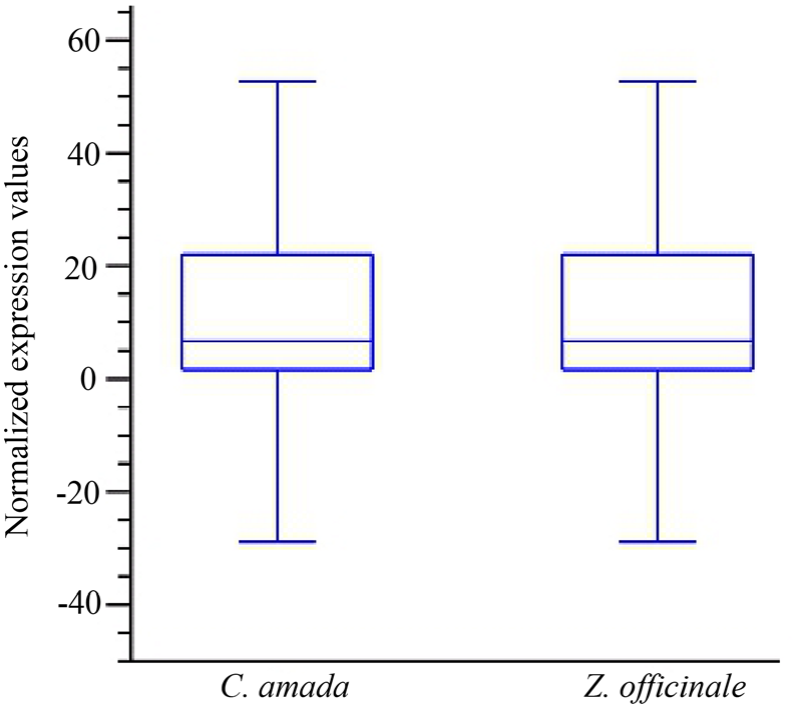
Box plot analysis of read mapping data after quantile normalization in *C. amada* and *Z. officinale* transcriptome. The transcript expression values (RPKM) overall distribution and variability of two cDNA libraries/samples were similar, indicating that they were comparable for identification of differentially expressed genes (DEGs) at the transcriptome level.

**Figure 5:**
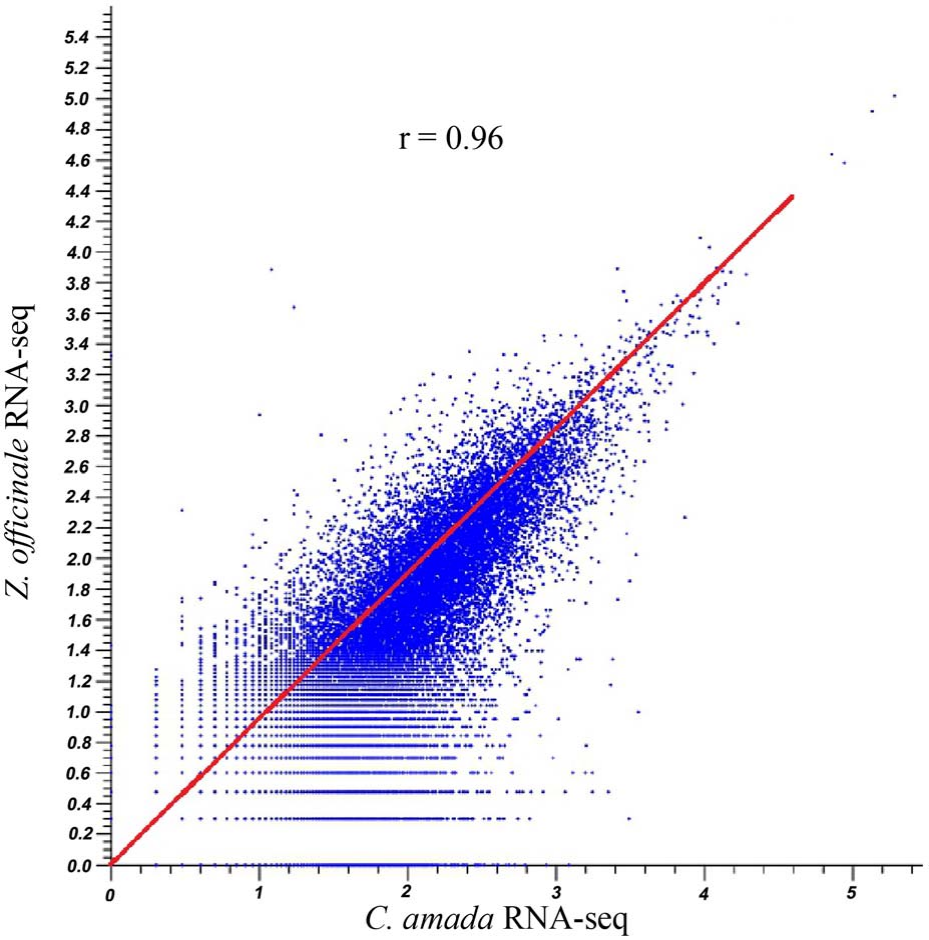
Pearson’s correlation coefficient between *C. amada* and *Z. officinale* transcriptomes. Comparisons of estimated RPKM distributions between *C. amada* and *Z. officinale* and *R. solanacearum* interactions. Pearson’s correlation coefficients (*R* ^2^) between transcriptomes are presented.

Analysis of differentially expressed transcripts between the *C. amada* and *Z. officinale* libraries should improve our understanding of the molecular events involved in disease resistance/susceptibility. The data on mapping reads to reference assembly (*C. amada de novo* assembly) was normalized by quantile normalization method. The boxplot of the normalized data clearly indicated matching variance and dispersion which statistically validate the downstream analysis of differential expression in these two species (Figure 4). To determine whether RNA-Seq read mapping data for both species was correlated, a Pearson’s correlation analysis was performed. The r value 0.96 indicated read mapping data is highly correlated (Figure 5). This confirms that the mapping of *Z. officnale* and *C. amada* read to *C. amada de novo* assembly was valid to find out differentially expressed genes in response to *R. solanacearum*.

**Figure 6:**
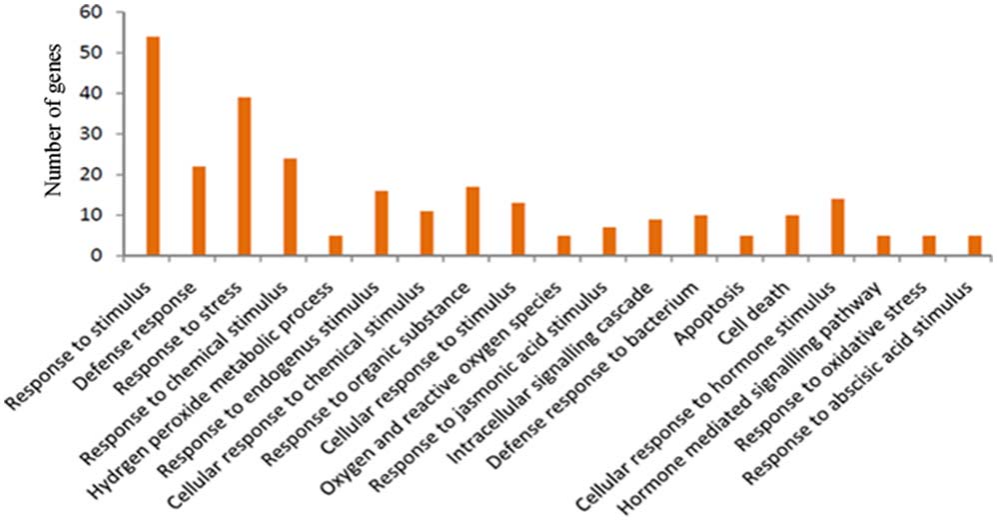
Histogram showing the significantly enriched Gene Ontology terms associated with up regulated genes in *C. amada*. The y-axis indicates the number of genes in a category.

A transcriptome comparison between *C. amada* and *Z. officinale* was performed to determine the effect of the infection by the *R. solanacearum* on gene expression. This comparison showed that the distribution of gene functions was generally similar in both the species. A total of 31385 *C. amada* and 20393 *Z. officinale* genes were expressed. The differential expression analysis using either RPKM or count data produced similar results. Based on 3 fold change and FDR *P* value<0.005 total of 1883 genes were identified as differentially expressed, out of which 560 genes were upregulated and 1234 genes were down regulated in *C. amada* (Table S1). We further grouped the differentially expressed genes into several functional categories on the basis of defense response, pathways and molecular function with respect to bacterial infection (Table 2, Table S2). A singular enrichment analysis of GO terms also revealed that defense related GO terms were significantly enriched at P<0.005 in *C. amada* (Figure 6).

**Table 2:** Summary of genes with high expression levels in response to *R. solanacearum* in *C. amada,* listed according to function.

Gene family	Up-regulated* in *C. amada*
Defense related	10
R-gene	2
Response to bacterium	6
Oxidative stress	7
Oxidation reduction	44
Jasmonic acid signaling	5
Ethylene signaling	1

*Fold-change between *C. amada* and *Z. officinale*, using fold-regulation cutoff of >3.0, *P*<0.005, FDR *P* at 0.05%.

**Figure 7:**
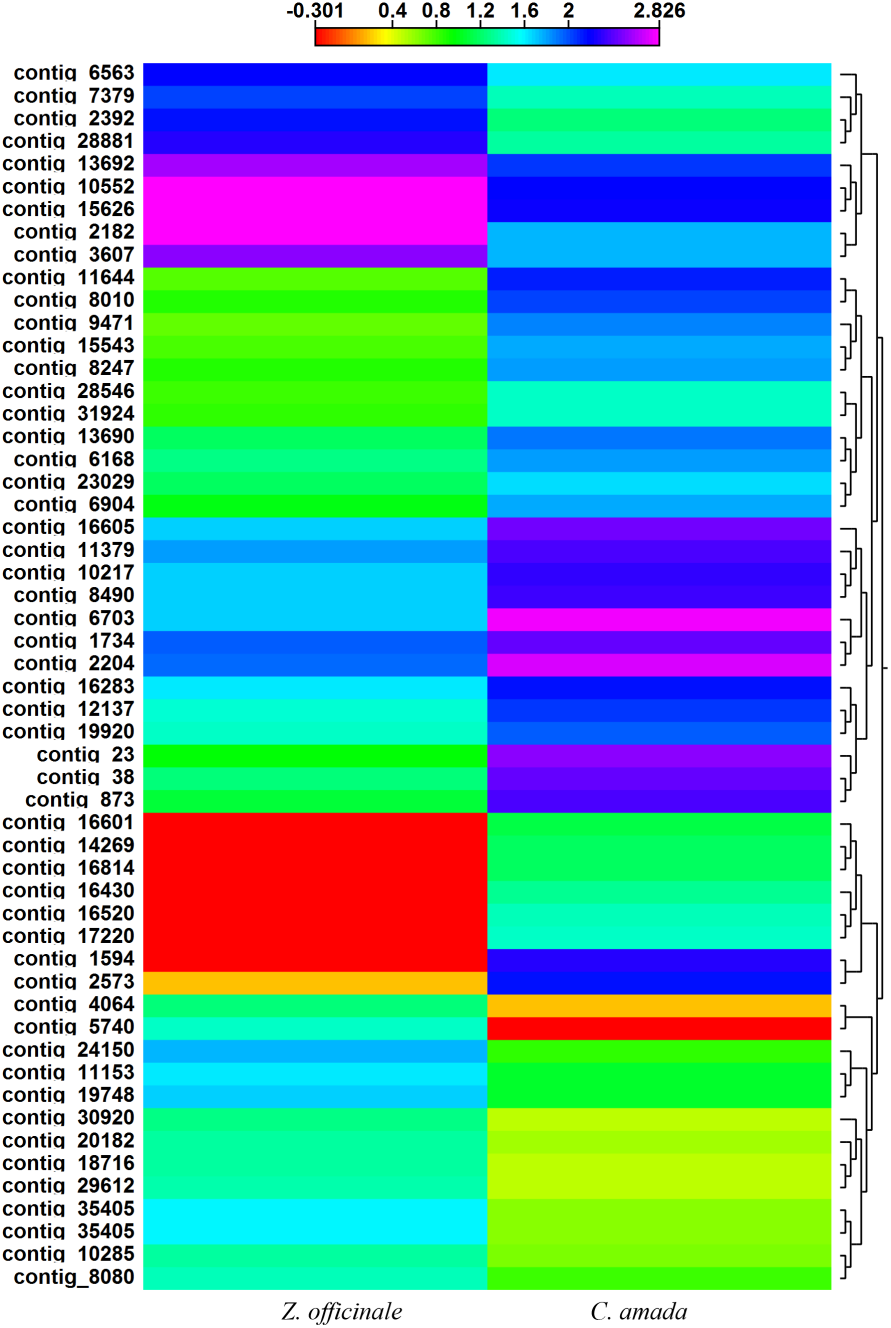
Divergence in the expression levels of transcription factors (TF) transcripts between C. amada and Z. officinale. Heat map and clustering analysis of the transcripts of genes encoding TFs are shown. Higher expression of TFs occurs in *C. amada*. The high expression levels are depicted in purple and low expression in red. Clustering and heat map was drawn with CLC Genomics Workbench based on an Euclidean distance matrix, after normalization of expression values.

Among the up regulated genes, we found 105 genes expressed only in *C. amada* in response to infection by *R. solanacearum*. Most important amongst these genes were fructose-bisphosphate aldolase activity, tropine dehydrogenase activity, resistance gene candidate NBS-type protein, phosphoenolpyruvate carboxykinase, heat shock protein 70, mannitol dehydrogenase, RGC3 resistance gene candidate NBS-type protein, CDPK-related protein kinase, disease resistance protein RPM1-like and zinc finger (C3HC4-type RING finger) protein-like, which were directly related to defense against pathogen through SA mediated hypersensitive, systemic acquired and cell death responses (Table 3, Table S3). Among the 54 differentially expressed transcription factors, 32 were up regulated in *C. amada,* which includes WRKY, MYB, leucine zipper protein, zinc finger and GATA domain transcription factors (Table 4, Table S4). Differential expression pattern of transcription factor genes is exhibited in figure 7.

**Table 3:** List of upregulated genes unique to *C. amada* during infection with *R. solanacearum.*

Gene ID	Functional annotation	Fold change*	*P*	FDR *P*
gi|639722|gb|AAA61682.1| calcium-dependent protein kinase	calcium-dependentprotein kinase	89.22	0.00E+00	0.00E+00
gi|224482647|gb|ACN50180.1| enolase	enolase	73.00	0.00E+00	0.00E+00
gi|211906504|gb|ACJ11745.1| heat shock protein 70	heat shock protein 70	61.10	0.00E+00	0.00E+00
gi|165967914|gb|ABY75803.1| resistance gene candidateNBS-type protein	resistance gene candidateNBS-type protein	42.50	3.13E−10	2.21E−09
gi|60657598|gb|AAX33320.1| secondary cell wall-relatedglycosyltransferase family 47	secondary cell wall-relatedglycosyltransferase family 47	45.50	6.76E−11	5.07E−10
gi|225429646|ref|XP_002281111.1| PREDICTED: thermosperminesynthase ACAULIS5	thermospermine synthaseACAULIS5	368.00	0.00E+00	0.00E+00
gi|225458880|ref|XP_002283423.1| PREDICTED: 70 kDapeptidyl-prolyl isomerase	70 kDa peptidyl-prolylisomerase	128.50	0.00E+00	0.00E+00
gi|38492172|gb|AAR22388.1| ANT-like protein	ANT-like protein	132.50	0.00E+00	0.00E+00
gi|242085822|ref|XP_002443336.1| hypothetical proteinSORBIDRAFT_08g017740	carboxylesterase activity	115.50	0.00E+00	0.00E+00
gi|224108908|ref|XP_002333333.1| predicted protein	fructose-bisphosphate aldolaseactivity	3901.00	0.00E+00	0.00E+00
gi|224056583|ref|XP_002298922.1| predicted protein	hydroxyacylglutathionehydrolase activity	218.00	0.00E+00	0.00E+00
gi|115463087|ref|NP_001055143.1| Os05g0304600	Lipoxygenase	137.00	2.22E−16	2.51E−15
gi|8118507|gb|AAF73006.1|AF262997_1 NADP-dependentmalic protein	malate dehydrogenase(oxaloacetate-decarboxylating)activity	106.00	0.00E+00	0.00E+00
gi|224137878|ref|XP_002322674.1| predicted protein	protein serine/threonine kinaseactivity	196.00	0.00E+00	0.00E+00
gi|226509426|ref|NP_001149968.1| LOC100283595	pyridoxine:NADP 4-dehydrogenaseactivity	78.00	9.02E−10	6.11E−09
gi|209167918|gb|ACI41983.1| putative pyruvate decarboxylase 1	pyruvate decarboxylase activity	55.75	0.00E+00	0.00E+00
gi|79314589|ref|NP_001030828.1| RING/U-box domain-containingprotein	RING/U-box domain-containingprotein	129.00	2.00E−15	2.13E−14
gi|297746005|emb|CBI16061.3| unnamed protein product	tropine dehydrogenase activity	271.00	0.00E+00	0.00E+00
gi|222637669|gb|EEE67801.1| hypothetical protein OsJ_25542	ubiquitin-protein ligase activity	79.00	0.00E+00	0.00E+00

*Fold-change between *C. amada* and *Z. officinale*, using fold-regulation cutoff of >40.0, *P*<0.005, FDR *P* at 0.05%.

**Table 4:** Summary of transcription factor unigenes of *C. amada* and *Z. officinale.*

Transcription Factors family	Number of genes detected	Up-regulated in *C. amada*	Up-regulated in *Z. officinale*
WRKY	8	4	4
MYB	6	4	2
AP2/ERF	2	2	-
MYC	1	1	-
GRAS	1	1	8
Zinc finger	17	9	1
bHLH	1	−	1
bZIP	3	2	4
Others	15	11	16
Total	54	34	20

### miRNA Target Prediction

Identification of miRNA target genes is a fundamental step for the determination of biological function for miRNAs. After carefully considering the alignment results, we located a total of 192 potential miRNA targets on *C. amada* and *Z. officinale* (Table S5). Annotation results of these transcripts further revealed that several defence related genes have miRNA targets as depicted in the Table S5. There were six and five targets predicted for miR169 and miR399, respectively, while the targets associated with other miRNAs were much less abundant. miRNAs with a large number of targets may represent nodes in gene regulatory networks, while those with a small number of targets may act through more specialized pathways. Other miRNA targets and respective miRNAs predicted in this study can be considered putative regulators of defense gene expression at the protein level.

### Ginger Transcriptome Database

We developed a public data resource, the ginger transcriptome database (gTDB), which provides a searchable interface to the transcriptome data. gTDB is publicly available at http://220.227.138.212/GTDB/. The current release (release 1.0) of the database provides the transcriptome sequence of the two species (*Curcuma amada* and *Zingiber officinale*) reported in this study. The web pages provide overview of the transcriptome sequencing, research group, and other resources of ginger transcriptome data. The database can be queried based on transcript ID and keyword search for all the functional annotations. The data available in the database can be mined using a variety of search options provided. The sequence-based search has been employed using NCBI BLASTsearch, which provides the option of different BLAST algorithms to search nucleotide or protein sequence(s) against ginger transcriptome sequence data reported in this study at user-defined parameters. The outputs include Annotation-Uniprot/Interproscan and GO Annotations. A link for downloading FASTA sequence of selected ID has been included. Various annotation search facilities are provided, namely, identifier search, gene ontology search and key word search (Figure S2).

## Discussion

Advances in DNA sequencing technology during the last decade have dramatically impacted genome sequencing and transcriptome analysis. Techniques such as microarrays and SAGE have facilitated transcriptome analysis at large scale from numerous plants. However, those techniques could be used only for model plants with known genome sequences. EST sequencing has been successfully used to analyze the transcriptome in non model plants. However, deep EST sequencing using capillary sequencing, which requires cDNA cloning and individual DNA preparations for each clone is time consuming and costly. Transcriptome analysis using Illumina sequencing technology is one of the most popular tools for gene discovery and it has been applied recently to several non-model species that lack genomic sequence information [38]–[41]. The results of the current study also indicated that relatively short reads from Illumina sequencing can be effectively assembled and used for novel gene discovery.

Before this work, only a few hundred ginger sequences had been deposited in the EST database (dbEST) at NCBI. The data presented here represent the first large effort by the PHYTOFURA project (a ICAR network project) to generate cDNA resources and analyze the transcriptomes of *C. amada* and *Z. officinale*. These resources are public and the sequences can be accessed in a searchable database. In total, our study generated 6.43 Gb and 4.87 Gb and tagged 36359 and 32312 transcripts from *C. amada* and *Z. officinale*, respectively. The two sets of unigenes from *C. amada* and *Z. officinale* also include a large number of genes known to be involved in response to general biotic and abiotic stimuli and stress. These gene sequences constitute a very important resource to the scientific community working on ginger wilt resistance as well as those interested in gene discovery in Zingiberaceae species. The cDNA sequences generated from both species cover various biological processes and molecular functions, indicating that Illumina sequencing constitutes a powerful tool for the transcriptome sequencing, characterization, and gene discovery of non model species.

GO annotation analyses showed that, in general, both the species have a similar transcriptome. Gene function categories associated with response to bacterium, wounding, stimulus, and signalling pathways were highly represented in both the species. The category represented the most was composed of genes associated with various transferase, transporter and reductase activities, as previously described in tomato [42].

Plants deploy a battery of mechanisms to defend themselves against pathogen infection. They have evolved complex defense strategies that include both constitutive and pathogen-induced components [43]. The front line of induced defense is triggered by pathogen-associated molecular patterns (PAMPs), also known as PAMP-triggered immunity. PAMPs are generally conserved compounds, as chitin in fungi and flagellins in bacteria, and PAMP-triggered immunity, which are induced by all invading pathogens [44]–[46]. The second line of plant defense is activated via recognition of pathogen effectors by resistance gene products followed by activation of effector-triggered immunity.

Detailed analysis of the sequences from both species showed that the tagged genes included a large number associated with resistance to biotic and abiotic stresses. These include number of genes involved in pathogen recognition and signaling, transcription factors, and resistance genes. Comparison of highly expressed genes showed that a fraction was either preferentially expressed in *C. amada* or in *Z. officinale*. The major functional categories observed in the *C. amada* and *Z. officinale* was represented by resistance proteins, response to bacterium, oxidative stress, oxidation reduction, ethylene (ET), jasmonic acid (JA) signalling and transcription factors. Such genes may modulate the expression of resistance genes in response to the bacterial infection. Among genes that were found to be differentially expressed in *C. amada*, several are known to be involved in various processes of plant defense against pathogens such as genes coding for defense response to bacterium, oxidative stress, oxidation reduction, construction of a physical barrier to block the pathogen progression, and systemic resistance.

The category of genes that seems to be involved in *C. amada* resistance to the bacterium encodes proteins involved in lignin biosynthesis such as cytochrome P450, succinyl-CoA ligase and S-adenosylmethionine synthase 1. Previous studies [47]–[49] showed that genes involved in lignin synthesis were over-expressed in various plants when they challenged with pathogens. These results suggest that the interaction between *C. amada* and *R. solanacearum* induces lignin accumulation, probably as a physical barrier.

Among other resistance genes over-expressed in *C. amada*, we found several phenylpropanoid pathway genes. Polyphenol oxidases (PPO), catalyzing the oxygen dependent oxidation of phenols to quinines have been demonstrated to increase tomato plant resistance against *Pseudomonas syringae* [50]. *ATPase* was found to be elevated in mango ginger. This gene is required for the attenuation of the hypersensitive response [50].

Transcriptome data in this paper suggest that hormone signaling is involved in the defense response. Among genes involved in signaling, we found several genes such as *mitogen activated protein*. This protein kinase activates both local resistance and basal resistance [51]. The expression level of β-cyanoalanine synthase gene was increased in response to *R. solanacearum* in *C. amada*. Consistent with these results, the silencing of genes involved in ET signaling transduction pathways caused breakdown of quantitative resistance against *R. solanacearum* [52]. In the present study also ERF transcription factor genes were also induced in *C. amada*. It is possible that they participate in activation of PR gene expressions [53].

Among transcription factor genes, 54 were found to be differentially expressed. Several transcription factors involved in the regulation of resistance gene expression such as bZIP, *WRKY*, *Zinc finger*, *Myb* etc were identified. In our transcriptome analysis, *Zinc finger*, *Myb, WRKY, ERF* and *MYC* factors easily dominated other classes of transcription factors in *C. amada*. *WRKY* transcription factors have been shown to fine tune the response of plants to challenge with pathogens [54], [55]. *Myb* genes are involved in regulation of disease resistance genes; they regulate the expression of *PAL2* gene, a key enzyme in phenylpropanoid and lignin biosynthesis [56].

Genes involved in mevalonate (MEP) pathway for biosynthesis of isoprene/terpenes have been found to be upregulated substantially in *C. amada* compared to *Z. officinale.* Several fold increase in the expression of fructose-bisphosphate aldolase ensure high production of pyruvate and D-glyceraldehyde 3-phosphate. The upregulated gene 1-D-deoxyxylulose 5-phosphate synthase converts glyceraldehydes 3-phosphate and pyruvate into 1-deoxy-D-xylulose 5-phosphate (DXP) [57], [58]. In the second step, DXP is converted to MEP by the enzyme DXP reductoisomerase (DXR) [59]–[62]. Similarly, the ATP-dependent 4-diphosphocytidyl-2*C*-methyl-Derythritol (CDP-ME) kinase (EC 2.7.1.148) that participates in the biosynthesis of isopentenyl diphosphate (IPP) and dimethylallyl diphosphate (DMAPP) has been found to exhibit many fold upregulation in *C. amada* compared to *Z. officinale* [63]. The upregulated enzyme 4-hydroxy-3-methylbut-2-en-1-yl diphosphate synthase in *C. amada* converts 2-C-methyl-D-erythritol-2,4-cyclodiphosphate into 1-hydroxy-2-methyl-2-(E)-butenyl 4-diphosphate in the methylerythritol phosphate pathway and it requires an intact [4Fe-4S] cluster for full activity.

There are eight consecutive enzyme steps in MEP pathway that produces isopentenyl diphosphate (IPP) and dimethylallyl diphosphate (DMAPP), the universal blocks of isoprenoid entities from the precursors pyruvate and D-glyceraldehyde 3-phosphate [64]. Of these eight enzymes, five enzymes involved in MEP pathway have shown many fold increase in *C. amada* compared to *Z. officinale,* except sesquiterpene synthase 5 which is down regulated indicating that cytosolic synthesis of sesquiterpene was not activated. All other up regulated enzymes indicated plastid synthesis of other terpenes through methylerythritol phosphate pathway (Table 5). The role of DXP in biosynthesis of IPP and DMAPP has been shown to be very critical [64]. This finding has been further substantiated form the observations that variation in the levels of DXP (either higher or lower) in *Arabidopsis* transgenic lines is reflected in higher or lower levels of isoprenoids final products correspondingly [65]. Similar results have been reported as in cases of tomato [66], potato [67] and *Ginkgo biloba* [68]. As there is a correspondence between the synthesis of DPP and IPP to the accumulating levels of various isoprenoids, it seems that DXP has a rate limiting function as reported in eubacteria [64].

**Table 5:** List of upregulated isoprene/terpene biosynthesis genes in *C. amada.*

Protein name	Fold change	GO functions
Fructose-bisphosphate aldolase(EC 4.1.2.13)	3901.00	GO:0006098 pentose-phosphate shunt; GO:0015976 carbon utilization
1-D-deoxyxylulose 5-phosphate synthase	10.36	GO:0016114 terpenoid biosynthetic process; GO:0006694 steroid biosynthetic process
4-diphosphocytidyl-2-C-methyl-Derythritol kinase	34.25	GO:0006694 steroid biosynthetic process
1-deoxy-D-xylulose 5-phosphatereductoisomerase	17.50	GO:0016114 terpenoid biosynthetic process; GO:0019288 isopentenyl diphosphate biosynthetic process, mevalonate-independent pathway
4-hydroxy-3-methylbut-2-en-1-yldiphosphate synthase	27.90	GO:0009862 systemic acquired resistance, salicylic acid mediated signaling pathway; GO:0019288 isopentenyl diphosphate biosynthetic process, mevalonate-independent pathway; GO:0009617 response to bacterium
Terpene synthase activity	3.03	GO:0000287 magnesium ion binding; GO:0010333 terpene synthase activity
2-C-methyl-D-erythritol 2,4cyclodiphosphate synthase	1.87	GO:0016114 terpenoid biosynthetic process
1-hydroxy-2-methyl-2-(E)-butenyl 4-diphosphate reductase	2.55	GO:0016114 terpenoid biosynthetic process; GO:0019288 isopentenyl diphosphate biosynthetic process, mevalonate-independent pathway

Terpenoids are the major group of volatile compounds in plants. There are several reports regarding antimicrobial activity of mango ginger extract [69]. Resistance of *C. amada* to *R. solanacearum* could be attributed to secretion of terpenoids and phenolic compounds as is reflected by the several fold upregulation of enzymes of MEP pathway leading to higher rate of terpenoid synthesis and secretion.

The predicted target genes in *C. amada* and *Z. officinale* had wide range of biological functions, and many encode transcription factors (BHLH, Zinc finger, GRAS, MYC, and WRKY transcription factor) and defense genes (NBS-LRR, β-1,3-glucanase, cytochrome P450, and S-adenosylmethionine synthase). miRNA was shown to function particularly during biotic and abiotic stresses with a multitude of miRNA up- or down-regulated during stress responses [70]. It also play an important role in stress tolerance by regulating processes such as hormone balance, transcription factors and defense genes [71], [72]. This study identified miRNA targets from *C. amada* and *Z. officinale*, thus representing a foundation for further research in identifying putative miRNAs and its role into transcriptional regulation of defense response.

In conclusion, this study allowed us to (i) Obtain over 36,000 and 32,000 unigenes from *C. amada* and *Z. officinale*, (ii) Compare the transcriptomes of mango ginger and ginger following infection by *R. solanacearum*, and (iii) Identify several candidate genes for resistance to bacterial wilt pathogen in mango ginger.

## Supporting Information

Figure S1 **Comparison of the percentage of** ***C. amada*** **and** ***Z. officinale*** **contigs that have best hits on the proteome of each of model species.**
(TIF)

Figure S2 **Snapshots of the public access resource gTDB showing its various utilities.**
(TIF)

Table S1 **List of genes differentially expressed in** ***C. amada*** **and** ***Z. officinale*** **along with their expression values.**
(XLS)

Table S2 **List of functional categories of differentially expressed genes in in** ***C. amada*** **and** ***Z. officinale*** **along with their expression values.**
(XLS)

Table S3 **List of upregulated genes unique to** ***C. amada*** **during infection with** ***R. solanacearum*** **.**
(XLS)

Table S4 **Transcription** **factors that showed differential expression levels in** ***C. amada*** **and** ***Z. officinale.***
(XLS)

Table S5 **miRNA potential target genes in C. amada and Z. officinale.**
(XLS)
