# Supplementary figures and images for: Comparison of the Transcriptomes of Ginger (Zingiber officinale Rosc.) and Mango Ginger (Curcuma amada Roxb.) in Response to the Bacterial Wilt Infection
Source: PLoS One. 2014 Jun 18;9(6):e99731. doi: 10.1371/journal.pone.0099731 (PMC4062433; doi:10.1371/journal.pone.0099731)

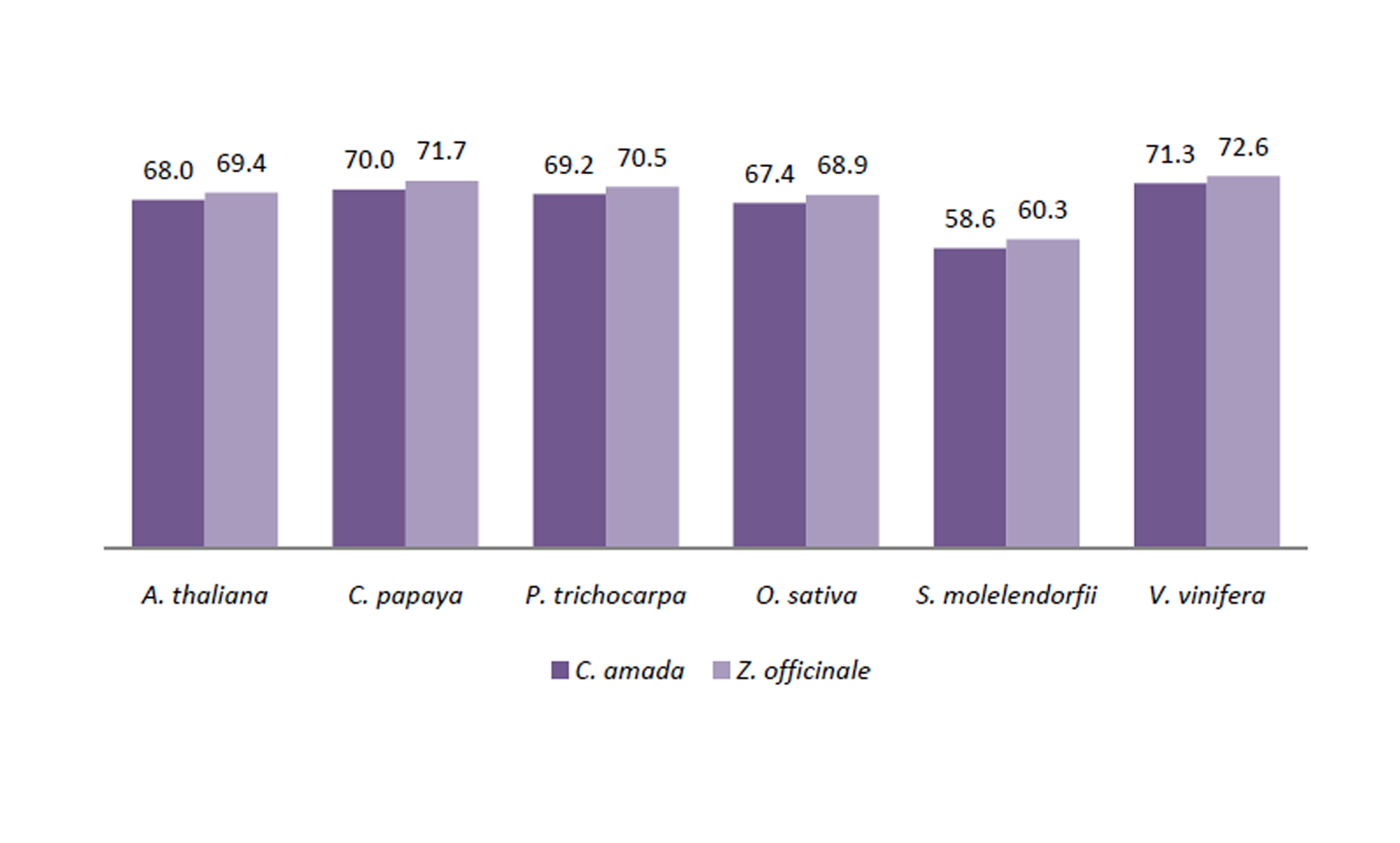

Supplement: Figure S1 — Comparison of the percentage of C. amada and Z. officinale contigs that have best hits on the proteome of each of model species. (TIF) [file pone.0099731.s001.tif]

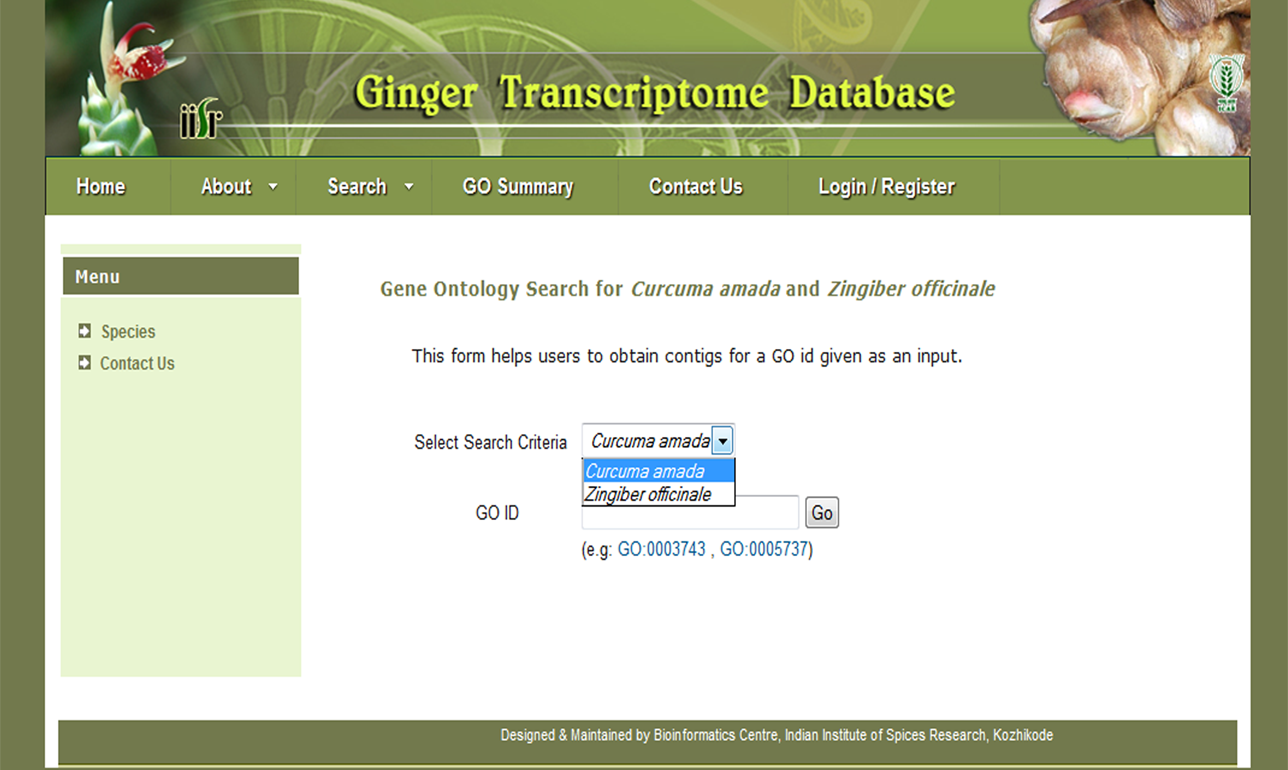

Supplement: Figure S2 — Snapshots of the public access resource gTDB showing its various utilities. (TIF) [file pone.0099731.s002.tif]
